# Maladaptive Plasticity in Levodopa-Induced Dyskinesias and Tardive Dyskinesias: Old and New Insights on the Effects of Dopamine Receptor Pharmacology

**DOI:** 10.3389/fneur.2014.00049

**Published:** 2014-04-09

**Authors:** Antonio Cerasa, Alfonso Fasano, Francesca Morgante, Giacomo Koch, Aldo Quattrone

**Affiliations:** ^1^Institute of Molecular Bioimaging and Physiology, National Research Council, Catanzaro, Italy; ^2^Movement Disorders Center, Division of Neurology, Toronto Western Hospital, University Health Network, University of Toronto, Toronto, ON, Canada; ^3^Dipartimento di Neuroscienze, Scienze Psichiatriche e Anestesiologiche, Università di Messina, Messina, Italy; ^4^Laboratorio di Neurologia Clinica e Comportamentale, Fondazione Santa Lucia IRCCS, Rome, Italy; ^5^Institute of Neurology, University “Magna Graecia”, Catanzaro, Italy

**Keywords:** levodopa-induced dyskinesias, tardive dyskinesias, hyperkinetic movement disorders, inferior frontal cortex, dopaminergic treatment

## Abstract

Maladaptive plasticity can be defined as behavioral loss or even development of disease symptoms resulting from aberrant plasticity changes in the human brain. Hyperkinetic movement disorders, in the neurological or psychiatric realms, have been associated with maladaptive neural plasticity that can be expressed by functional changes such as an increase in transmitter release, receptor regulation, and synaptic plasticity or anatomical modifications such as axonal regeneration, sprouting, synaptogenesis, and neurogenesis. Recent evidence from human and animal models provided support to the hypothesis that these phenomena likely depend on altered dopamine turnover induced by long-term drug treatment. However, it is still unclear how and where these altered mechanisms of cortical plasticity may be localized. This study provides an up-to-date overview of these issues together with some reflections on future studies in the field, particularly focusing on two specific disorders (levodopa-induced dyskinesias in Parkinson’s disease patients and tardive dyskinesias in schizophrenic patients) where the modern neuroimaging approaches have recently provided new fundamental insights.

## Introduction

Plasticity refers to the ability of the nervous system to change the effectiveness of transmission in neural circuits. This can involve changes at several levels (neuronal, synaptic, protein, or genomic structure) and modulates both the structure and function of neuronal networks. Several human and animal studies demonstrated that exercise and/or behavioral enrichment can increase neuronal survival and resistance to brain insult, promote brain vascularization, stimulate neurogenesis, and enhance learning [for review, see Ref. (1)].

Although neural plasticity is generally viewed as an adaptive process, there is considerable evidence that plasticity can also be maladaptive [for review, see Ref. (2)]. For instance, sensory deprivation, chronic stress, and excessive exercise would reduce variability and impair adaptability. In particular, chronic stress is associated with a loss of neurons and synapses. Furthermore, stress may increase activity in certain brain regions, such as the amygdala and the mesolimbic dopaminergic system, leading to hypertrophy of these structures (3). Again, Byl et al. (4) showed that monkeys that were over-trained to make a particular highly specific hand movement sometimes developed difficulties in moving their hands in a similar manner to focal hand dystonia. The somatosensory cortex of these animals was less organized than that of healthy monkeys, with larger receptive fields and overlapping representations of the individual digits. A change in the pattern of connectivity in the sensory and motor cortices was thought to lead to inappropriate associations between inputs and outputs of the motor areas and cause errors in selecting muscles used in voluntary movement.

However, maladaptive neural plasticity may be triggered not only by exercise and/or behavioral deprivation, but also by other factors, such as chronic drug therapy. In the last few years, several influential authors (5–7) proposed that some hyperkinetic movement disorders [levodopa-induced dyskinesias (LIDs), primary dystonia, Huntington’s disease, and tardive dyskinesias (TDs)] are caused by maladaptive synaptic plasticity. The scope of this study is to summarize evidence on the role of dopaminergic replacement in inducing maladaptive neural changes in these hyperkinetic disorders, delineating the presence of shared neural mechanisms. Particular attention will be paid to recent evidence coming from molecular and neuroimaging studies that allow *in vivo* evaluation of neural plasticity.

## Parkinson’s Disease with Levodopa-Induced Dyskinesias

The classical clinical picture of Parkinson’s disease (PD) consists of motor deficits, such as akinesia, rigidity, tremor, and postural dysfunction. These motor symptoms are greatly improved by treatments with dopamine (DA) replacement therapy or DA agonists, but after 4–6 years, the therapeutic window becomes narrow and patients start to experience very disabling motor symptoms, such as motor fluctuations and LIDs. LIDs in PD has been thought to originate from an imbalance between “direct” and “indirect” pathways regulating neural activity in the striato-frontal network (8, 9). The neurons of the direct pathway project to the globus pallidus pars interna (GPi) and onward to the thalamus. The neurons comprise the indirect pathway project to the globus pallidus pars externa (GPe), where they synapse with more GABAergic projection neurons. In turn, these neurons project to the subthalamic nuclei (STN) and form synapses with the glutamatergic neurons that provide output to the GPi and the substantia nigra pars reticulata (SNr). The essential pathophysiological characteristic of the LIDs state is the presence of under-activity of the indirect pathway and overactivity of the direct pathway. In the last few years, this model has been reinforced and modified to extend knowledge about the interplay between striatal nuclei and the frontal cortex. Indeed, LIDs have also been demonstrated to be associated with a sequence of events that includes pre-synaptic (i.e., increased synaptic level of DA) and post-synaptic modifications (i.e., downstream changes in proteins and genes), and abnormalities in non-DA transmitter systems (9). Overall, all these events combine to produce alterations in the firing patterns and the coherence between the basal ganglia and the cortex, leading to excessive disinhibition of thalamocortical neurons and overactivation of frontal areas, with specific involvement of motor, premotor, and prefrontal cortices (10, 11). The presence of altered cortical excitability in the motor and prefrontal cortex has also been demonstrated by electrophysiological studies employing transcranial magnetic stimulation (TMS) in patients with PD (12, 13). Moreover, as repetitive TMS applied over the supplementary motor area (SMA), the primary motor cortex (M1) was able to induce a transient reduction of LIDs severity (14, 15). Despite the traditional striato-thalamo-cortical pathways, in the last few years, advances in the neurophysiological field provide alternative scenarios highlighting the involvement of other circuits involved in the pathophysiological mechanisms of LIDs. In particular, reduction of peak-dose dyskinesia for up to 4 weeks was described following repeated sessions of continuous theta burst stimulation (cTBS) delivered bilaterally to the lateral cerebellum (16). This later finding would seem to support the hypothesis that alterations in cerebellar sensory processing function, occurring secondary to abnormal basal ganglia signals reaching it, may be an important element contributing to the maladaptive sensorimotor plasticity of motor cortex and the emergence of abnormal involuntary movements (17).

## Tardive Dyskinesias in Psychiatric Disorders

Chronic blockage of DA receptors by anti-psychotic drugs in patients with psychiatric disorders has been known to produce another well-known hyperkinetic movement disorder named TDs. Originally, the term TDs referred to abnormal movements produced by long-term DA receptor antagonist therapy, mainly characterized by rapid, repetitive, stereotypic movements affecting mainly the oral, buccal, and lingual areas, and less movements affecting the limb and the trunk; however, other phenomenologies have been described in TDs, such as dystonia and akathisia (18). More than half of TDs cases may persist, even after conventional antipyschotics are switched to atypical ones (19), or anti-psychotics are discontinued (20). The most popular pathophysiological model for TD is based on DA receptor hypersensitivity triggered by anti-psychotic drugs (7, 21, 22). According to this theory, chronic use of DA antagonists, particularly at high levels of antagonism (i.e., haloperidol), results in gradual hypersensitization of DA receptors. Indeed, chronic administration of neuroleptics might cause adaptive changes in DA receptors, causing an increase in the number of D2 receptors (23). D2 receptors being expressed on indirect pathway medium-spiny neurons and being inhibitory, the consequence of D2 hypersensitivity might determine disinhibition of the GPi and the subthalamic nucleus (7). Support for hypersensitivity of DA receptors in TDs mainly comes from rodent models (24). Moreover, further support comes from clinical observations that increasing anti-psychotic dosage temporarily suppresses TD (25) whereas withdrawing anti-psychotics or administering DA agonists exacerbates dyskinetic symptoms in the short term (21). However, although chronic anti-psychotic use is associated with D2 hypersensitivity, the evidence supporting a direct role on TDs generation are not consistent; indeed, DA receptors binding in PET studies was not correlated with severity of dyskinesias and post-mortem studies did not disclose any difference in number of D2 receptors between patients with and without TDs (26).

## Shared Maladaptive Neural Plasticity in LIDs and TDs Patients

Although LIDs and TDs are underlined by drugs acting on the DA receptor in an opposite way and associated with different diseases, there is evidence for shared neurodegenerative mechanisms, likely depending on aberrant neural plasticity in the prefrontal cortex.

The development of LIDs has been attributed in cell studies to dysfunctional cortico-striatal plasticity triggered by the combined effects of DA denervation and chronic pharmacological DA replacement (27) and has also been demonstrated *in vivo* in PD patients with LIDs (12, 13). Chronic non-physiologic stimulation of DA receptors on striatal neurons (23) can induce modifications in NMDA receptors firing and thus the development of aberrant motor patterns leading to motor complications. This has provided the rationale for the use of NMDA receptor antagonists such as amantadine for treating PD patients with LIDs (28). Despite the recent evidence on the efficacious effects of amantadine in ameliorating LIDs [improved motor symptoms in 60–70% of patients; (29), the restoring of deficient DA with its precursor l-3,4-dihydroxyphenylalanine (levodopa) is again the most effective treatment for PD. In the last decades, concern has been raised that levodopa could have toxic effects on the brain of patients with PD (30). Although some *in vitro* studies support this hypothesis (31), this concern remains uncertain in human studies (32). Recent evidence coming from neuroimaging studies has provided new impetus to unravel the potential effects of levodopa on brain morphometry. Indeed, our group has recently demonstrated that specific morphological alterations are associated with the development of LIDs in PD patients (33). Using unbiased voxel-based morphometry to compare gray matter volume in dyskinetic and non-dyskinetic patients, closely matched for age, duration of medication and age of onset, we observed significant increases of gray matter volume within the inferior frontal cortex (IFC), the degree of which increased with the severity of motor fluctuations. The IFC is an associative area specifically involved, together with motor and premotor cortices, basal ganglia and STN, in the regulation of motor inhibition (34). The presence of “increased” gray matter volume was in accordance with other neuroimaging studies investigating the neurobiological effects of levodopa treatment in healthy individuals. Indeed, Salgado-Pineda et al. (35) demonstrated an increased gray matter volume in healthy controls 1 week after levodopa administration. All this evidence suggested that levodopa applied in a pulsatile and non-physiological manner can perturb the normal physiological mechanisms that mediate motor control and eventually result in the remodeling of neuronal contacts and pathways, producing long-lasting changes and aberrant neural plasticity (i.e., increased neuronal arborization). The presence of altered anatomy of the IFC has been confirmed using different populations (36) and neuroimaging metrics [i.e., cortical thickness; Ref. (37)] and has raised an interesting scientific debate on the potential effects of levodopa on brain morphometry (38, 39). In particular, the main notion proposed by Vernon and Modo (38) concerned the key role that animal models of PD would play in providing new insight about the hypothesis proposed above. Indeed, although several models of basal ganglia dysfunctions have been proposed to understand the pathophysiological mechanisms underlying motor manifestations in patients with PD (8), these changes cannot completely explain the overall motor symptoms of LIDs in patients. Indeed, a large body of *in vitro* and *in vivo* studies in animal models of parkinsonism have suggested an alternative “glutamatergic” hypothesis for LIDs. Several authors demonstrated that the glutamatergic cortico-striatal projection to medium-spiny neurons might play an important role in the priming and development of LIDs, by induction of abnormal synaptic plasticity at the cortico-striatal level (40–42). The alteration of glutamatergic transmission as causative of LIDs has also been demonstrated *in vivo* by Ahmed et al. (43), who described abnormalities in glutamate transmission in striatal and frontal regions in dyskinetic compared to non-dyskinetic patients with PD. Despite the plethora of studies supporting the glutamatergic contribution to LIDs, there is also a vast consensus that dysfunctions of the serotonergic system are implicated in the development of LIDs and other complications of levodopa therapy. Indeed, serotonin neurons have the ability to synthesize, store, and release DA, formed from exogenous levodopa, but due to the lack of any autoregulatory feedback control, the DA released from serotonin terminals generally show excessive swings in the patients in response to repetitive, intermittent levodopa treatment (44). Such dysregulated release of levodopa-derived DA is likely to be the main trigger of dyskinesia in levodopa-primed animals (44), and may also play a role in PD patients undergoing long-term levodopa therapy (45, 46). Using animal models, Rylander et al. (47) provided the first evidence that levodopa treatment induces sprouting of serotonin axon terminals, with an increased incidence of synaptic contacts and a larger activity-dependent potentiation of DA release in the DA-denervated striatum. This latter finding is of great interest since our reported morphological abnormalities in patients with LIDs (33, 36) highlighted the role of the IFC, a region strongly innervated and regulated by the serotonergic system (48, 49).

Interestingly, similar evidence, highlighting the presence of neural abnormalities in the IFC driven by chronic DA therapy, has also been provided in the psychiatric realm. In particular, chronic psychotropic treatment might cause either adaptive changes in DA receptors (increase in the number of D2 receptors and supersensitivity of D1 receptors) (50) or determine structural remodeling of the brain. Post-mortem studies investigating brains of schizophrenic patients with long duration of anti-psychotic exposure (i.e., haloperidol) showed significant structural abnormalities (51–54), with evidence for slight shrinkage (5%) of the brain in terms of weight, length, and cortical volume and for enlarged (15%) ventricles. Animal studies have confirmed this evidence, demonstrating that chronic (8 weeks) exposure to both haloperidol and olanzapine resulted in significant decreases in whole-brain volume (6–8%) driven mainly by a decrease in frontal cerebral cortex volume (8–12%) (55). Finally, a recent *in vivo* neuroimaging study investigating the neuroanatomical differences between schizophrenic patients with TDs with respect to patients without TDs (closely matched for age at onset of illness, duration of illness, or anti-psychotic chlorpromazine equivalent dose) demonstrated, for the first time, the presence of volumetric abnormalities in the same prefrontal region described in patients with LIDs: the IFC. The merit of this work (56) was to provide evidence on the presence of maladaptive neural rearrangements in the IFC driven by chronic psychotropic treatment.

## Conclusion

Although all these findings are pieces of a very difficult puzzle to assemble, what clearly emerged is that all these disorders have been associated with altered DA turnover induced by long-term drug treatment that might ultimately induce maladaptive synaptic plasticity. We believe that further advances in the understanding of the maladaptive mechanisms of synaptic plasticity in other hyperkinetic movement disorders (Tourette syndrome, dystonia, and Huntington’s disease) will lead in the next few years to defining the exact biological impact of chronic DA therapy on the neurological and psychiatric brain, which might ultimately stimulate development for new treatments. For instance, in a recent neuroimaging study, Ganos et al. (57) described the presence of gray matter abnormalities of the IFC in patients affected by Tics in Gilles de la Tourette syndrome. So far, evidence emerging from recent molecular and neuroimaging studies would seem to suggest an intriguing hypothesis that some important hyperkinetic movement disorders might share similar pathophysiological mechanisms (7). In particular, part of these shared mechanisms would seem to be localized outside the classical motor pathway (cerebellum-striato-thalamic-motor network), involving a critical region (IFC) taking part in the hyperdirect pathway, a neural circuit playing a critical role in motor control (58), which might become a new potential therapeutic target for future studies (59).

## Conflict of Interest Statement

The authors declare that the research was conducted in the absence of any commercial or financial relationships that could be construed as a potential conflict of interest.

## References

[1] Castrén E Hen R Neuronal plasticity and antidepressant actions. Trends Neurosci (2013) 36(5):259–67 10.1016/j.tins.2012.12.010 23380665 PMC3648595

[2] Quartarone A Siebner HR Rothwell JC Task-specific hand dystonia: can too much plasticity be bad for you? Trends Neurosci (2006) 29(4):192–9 10.1016/j.tins.2006.02.007 16519953

[3] McEwen BS Stress and hippocampal plasticity. Annu Rev Neurosci (1999) 22:105–22 10.1146/annurev.neuro.22.1.105 10202533

[4] Byl NN Merzenich MM Jenkins WM A primate genesis model of focal dystonia and repetitive strain injury: I. Learning-induced dedifferentiation of the representation of the hand in the primary somatosensory cortex in adult monkeys. Neurology (1996) 47:508–20 10.1212/WNL.47.2.508 8757029

[5] Cenci MA Dopamine dysregulation of movement control in L-DOPA-induced dyskinesia. Trends Neurosci (2007) 30(5):236–43 10.1016/j.tins.2007.03.005 17400300

[6] Breakefield XO Blood AJ Li Y Hallett M Hanson PI Standaert DG The pathophysiological basis of dystonias. Nat Rev Neurosci (2008) 9(3):222–34 10.1038/nrn2337 18285800

[7] Teo JT Edwards MJ Bhatia K Tardive dyskinesia is caused by maladaptive synaptic plasticity: a hypothesis. Mov Disord (2012) 27(10):1205–15 10.1002/mds.25107 22865512

[8] Obeso JA Rodriguez-Oroz MC Rodriguez M Lanciego JL Artieda J Gonzalo N Pathophysiology of the basal ganglia in Parkinson’s disease. Trends Neurosci (2000) 23(10):S8–19 10.1016/S1471-1931(00)00028-8 11052215

[9] Jenner P Molecular mechanisms of L-DOPA-induced dyskinesia. Nat Rev Neurosci (2008) 9(9):665–77 10.1038/nrn2471 18714325

[10] Rascol O Sabatini U Brefel C Fabre N Rai S Senard JM Cortical motor overactivation in parkinsonian patients with levodopa-induced peak-dose dyskinesia. Brain (1998) 121(3):527–33 10.1093/brain/121.3.527 9549528

[11] Brooks DJ Piccini P Turjanski N Samuel M Neuroimaging of dyskinesia. Ann Neurol (2000) 47(4):S154–8 10762143

[12] Morgante F Espay AJ Gunraj C Lang AE Chen R Motor cortex plasticity in Parkinson’s disease and levodopa-induced dyskinesias. Brain (2006) 129(Pt 4):1059–69 10.1093/brain/awl031 16476674

[13] Kishore A Popa T Velayudhan B Joseph T Balachandran A Meunier S Acute dopamine boost has a negative effect on plasticity of the primary motor cortex in advanced. Parkinson’s disease. Brain (2012) 135(7):2074–88 10.1093/brain/aws124 22609619

[14] Koch G Brusa L Caltagirone C Peppe A Oliveri M Stanzione P rTMS of supplementary motor area modulates therapy-induced dyskinesias in Parkinson disease. Neurology (2005) 65:623–5 10.1212/01.wnl.0000172861.36430.95 16116131

[15] Koch G rTMS effects on levodopa induced dyskinesias in Parkinson’s disease patients: searching for effective cortical targets. Restor Neurol Neurosci (2010) 28(4):561–8 10.3233/RNN-2010-0556 20714078

[16] Koch G Brusa L Carrillo F Lo Gerfo E Torriero S Oliveri M Cerebellar magnetic stimulation decreases levodopa-induced dyskinesias in Parkinson disease. Neurology (2009) 73:113–9 10.1212/WNL.0b013e3181ad5387 19597133

[17] Kishore A Popa T Balachandran A Chandran S Pradeep S Backer F Cerebellar sensory processing alterations impact motor cortical plasticity in Parkinson’s disease: clues from dyskinetic patients. Cereb Cortex (2013). 10.1093/cercor/bht058 23535177

[18] Jankovic J Tardive syndromes and other drug-induced movement disorders. Clin Neuropharmacol (1995) 18(3):197–214 10.1097/00002826-199506000-00001 8635179

[19] Sigwald J Bouttier D Raymondeaud C Piot C [4 Cases of faciobucco-linguo-masticatory dyskinesis of prolonged development following treatment with neuroleptics]. Rev Neurol (Paris) (1959) 100:751–5 [Article in French]. 14446619

[20] Baldessarini RJ Tarsy D Mechanisms underlying tardive dyskinesia. Res Publ Assoc Res Nerv Ment Dis (1976) 55:433–46 826999

[21] Marsden CD Jenner P The pathophysiology of extrapyramidal side-effects of neuroleptic drugs. Psychol Med (1980) 10(1):55–72 10.1017/S003329170003960X 6104342

[22] Calabresi P De Murtas M Mercuri NB Bernardi G Chronic neuroleptic treatment: D2 dopamine receptor supersensitivity and striatal glutamatergic transmission. Ann Neurol (1992) 31(4):366–73 10.1002/ana.410310404 1350190

[23] Casey DE Tardive dyskinesia: pathophysiology and animal models. J Clin Psychiatry (2000) 61(Suppl 4):5–9 10739324

[24] Ananth J Current psychopathological theories of tardive dyskinesia and their implications for future research. Neuropsychobiology (1982) 8(4):210–22 10.1159/000117901 6127649

[25] Peralta V Campos MS De Jalon EG Cuesta MJ Motor behavior abnormalities in drug-naïve patients with schizophrenia. Mov Disord (2010) 25(8):1068–76 10.1002/mds.23050 20222137

[26] Crow TJ Cross AJ Johnstone EC Owen F Owens DG Waddington JL Abnormal involuntary movements in schizophrenia: are they related to the disease process or its treatment? Are they associated with changes in dopamine receptors? J Clin Psychopharmacol (1982) 2(5):336–40 10.1097/00004714-198210000-00010 7130435

[27] Calabresi P Giacomini P Centonze D Bernardi G Levodopa-induced dyskinesia: a pathological form of striatal synaptic plasticity? Ann Neurol (2000) 47:S60–8 10762133

[28] Gottwald MD Aminoff MJ Therapies for dopaminergic-induced dyskinesias in Parkinson disease. Ann Neurol (2011) 69(6):919–27 10.1002/ana.22423 21681795

[29] Sawada H Oeda T Kuno S Nomoto M Yamamoto K Yamamoto M Amantadine for dyskinesias in Parkinson’s disease: a randomized controlled trial. PLoS One (2010) 5(12):e15298 10.1371/journal.pone.0015298 21217832 PMC3013111

[30] Zesiewicz TA Parkinson disease: the controversy of levodopa toxicity in Parkinson disease. Nat Rev Neurol (2011) 8(1):8–10 10.1038/nrneurol.2011.199 22198404

[31] Spencer JP Jenner A Butler J Aruoma OI Dexter DT Jenner P Evaluation of the pro-oxidant and antioxidant actions of L-DOPA and dopamine in vitro: implications for Parkinson’s disease. Free Radic Res (1996) 24(2):95–105 10.3109/10715769609088005 8845917

[32] Fahn S Oakes D Shoulson I Kieburtz K Rudolph A Lang A Levodopa and the progression of Parkinson’s disease. N Engl J Med (2004) 351:2498–508 10.1056/NEJMoa033447 15590952

[33] Cerasa A Messina D Pugliese P Morelli M Lanza P Salsone M Increased prefrontal volume in PD with levodopa-induced dyskinesias: a voxel-based morphometry study. Mov Disord (2011) 26(5):807–12 10.1002/mds.23660 21384430

[34] Aron AR Robbin TW Poldrack RA Inhibition and the right inferior frontal cortex. Trends Cogn Sci (2004) 8(4):170–7 10.1016/j.tics.2004.02.010 15050513

[35] Salgado-Pineda P Delaveau P Falcon C Blin O Brain T1 intensity changes after levodopa administration in healthy subjects: a voxel-based morphometry study. Br J Clin Pharmacol (2006) 62:546–51 10.1111/j.1365-2125.2006.02695.x 16796705 PMC1885173

[36] Cerasa A Morelli M Augimeri A Salsone M Novellino F Gioia MC Prefrontal thickening in PD with levodopa-induced dyskinesias: new evidence from cortical thickness measurement. Parkinsonism Relat Disord (2013) 19(1):123–5 10.1016/j.parkreldis.2012.06.003 22742954

[37] Cerasa A Salsone M Morelli M Pugliese P Arabia G Gioia MC Age at onset influences neurodegenerative processes underlying PD with levodopa-induced dyskinesias. Parkinsonism Relat Disord (2013) 19(10):883–8 10.1016/j.parkreldis.2013.05.015 23769805

[38] Vernon AC Modo M Do levodopa treatments modify the morphology of the parkinsonian brain? Mov Disord (2012) 27(1):166–7 10.1002/mds.24018 22076912

[39] Aron AR Obeso J Is executive control used to compensate for involuntary movements in levodopa-induced dyskinesia? Mov Disord (2012) 27(3):339–40 10.1002/mds.24936 22411844

[40] Picconi B Pisani A Barone I Bonsi P Centonze D Bernardi G Pathological synaptic plasticity in the striatum: implications for Parkinson’s disease. Neurotoxicology (2005) 26(5):779–83 10.1016/j.neuro.2005.02.002 15927256

[41] Sgambato-Faure V Cenci MA Glutamatergic mechanisms in the dyskinesias induced by pharmacological dopamine replacement and deep brain stimulation for the treatment of Parkinson’s disease. Prog Neurobiol (2012) 96(1):69–86 10.1016/j.pneurobio.2011.10.005 22075179

[42] Cenci MA Konradi C Maladaptive striatal plasticity in L-DOPA-induced dyskinesia. Prog Brain Res (2010) 183:209–33 10.1016/S0079-6123(10)83011-0 20696322 PMC2930606

[43] Ahmed I Bose SK Pavese N Ramlackhansingh A Turkheimer F Hotton G Glutamate NMDA receptor dysregulation in Parkinson’s disease with dyskinesias. Brain (2011) 134(Pt 4):979–86 10.1093/brain/awr028 21371994

[44] Carta M Carlsson T Kirik D Bjorklund A Dopamine released from 5-HT terminals is the cause of L-DOPA-induced dyskinesia in parkinsonian rats. Brain (2007) 130(7):1819–33 10.1093/brain/awm082 17452372

[45] de la Fuente-Fernandez R Lu JQ Sossi V Jivan S Schulzer M Holden JE Biochemical variations in the synaptic level of dopamine precede motor fluctuations in Parkinson’s disease: PET evidence of increased dopamine turnover. Ann Neurol (2001) 49(3):298–303 10.1002/ana.65 11261503

[46] de la Fuente-Fernandez R Schulzer M Mak E Calne DB Stoessl AJ Presynaptic mechanisms of motor fluctuations in Parkinson’s disease: a probabilistic model. Brain (2004) 127(4):888–99 10.1093/brain/awh102 14960500

[47] Rylander D Parent M O’Sullivan SS Dovero S Lees AJ Bezard E Maladaptive plasticity of serotonin axon terminals in levodopa-induced dyskinesia. Ann Neurol (2010) 68(5):619–28 10.1002/ana.22097 20882603

[48] Clarke HF Dalley JW Crofts HS Robbins TW Roberts AC Cognitive inflexibility after prefrontal serotonin depletion. Science (2004) 304(5672):878–80 10.1126/science.1094987 15131308

[49] Cerasa A Cherubini A Quattrone A Gioia MC Magariello A Muglia M Morphological correlates of MAO A VNTR polymorphism: new evidence from cortical thickness measurement. Behav Brain Res (2010) 211(1):118–24 10.1016/j.bbr.2010.03.021 20303364

[50] Casey DE Pathophysiology of antipsychotic drug-induced movement disorders. J Clin Psychiatry (2004) 65(Suppl 9):25–8 15189109

[51] Crow TJ Ball J Bloom SR Brown R Bruton CJ Colter N Schizophrenia as an anomaly of development of cerebral asymmetry. A postmortem study and a proposal concerning the genetic basis of the disease. Arch Gen Psychiatry (1989) 46(12):1145–50 10.1001/archpsyc.1989.01810120087013 2589928

[52] Pakkenberg B Post-mortem study of chronic schizophrenic brains. Br J Psychiatry (1987) 151:744–752 10.1192/bjp.151.6.744 3502800

[53] Heckers S Neuropathology of schizophrenia: cortex, thalamus, basal ganglia, and neurotransmitter-specific projection systems. Schizophr Bull (1997) 23(3):403–21 10.1093/schbul/23.3.403 9327506

[54] Jellinger K The neuropathology of schizophrenia. J Neuropathol Exp Neurol (1999) 58(11):1192 10560662 10.1097/00005072-199911000-00009

[55] Vernon AC Natesan S Crum WR Cooper JD Modo M Williams SC Contrasting effects of haloperidol and lithium on rodent brain structure: a magnetic resonance imaging study with postmortem confirmation. Biol Psychiatry (2012) 71(10):855–63 10.1016/j.biopsych.2011.12.004 22244831

[56] Li CT Chou KH Su TP Huang CC Chen MH Bai YM Gray matter abnormalities in schizophrenia patients with tardive dyskinesia: a magnetic resonance imaging voxel-based morphometry study. PLoS One (2013) 8(8):e71034 10.1371/journal.pone.0071034 23967150 PMC3744521

[57] Ganos C Kühn S Kahl U Schunke O Brandt V Bäumer T Prefrontal cortex volume reductions and tic inhibition are unrelated in uncomplicated GTS adults. J Psychosom Res (2014) 76:84–7 10.1016/j.jpsychores.2013.10.014 24360147

[58] Nambu A Tokuno H Takada M Functional significance of the cortico-subthalamo-pallidal ‘hyperdirect’ pathway. Neurosci Res (2002) 43(2):111–7 10.1016/S0168-0102(02)00027-5 12067746

[59] Cerasa A Quattrone A May hyperdirect pathway be a plausible neural substrate for understanding the rTMS-related effects on PD patients with levodopa-induced dyskinesias? Brain Stimul (2014). 10.1016/j.brs.2014.01.007 24507575

